# Missed Opportunities: A Narrative Review on Why Nonoccupational Postexposure Prophylaxis for HIV Is Underutilized

**DOI:** 10.1093/ofid/ofae332

**Published:** 2024-06-15

**Authors:** Lao-Tzu Allan-Blitz, Kenneth H Mayer

**Affiliations:** Division of Global Health Equity, Department of Medicine, Brigham and Women's Hospital, Boston, Massachusetts, USA; Department of Medicine, Harvard Medical School, Boston, Massachusetts, USA; The Fenway Institute of Fenway Health, Boston, Massachusetts, USA; Fenway Health, Boston, Massachusetts, USA; Division of Infectious Diseases, Department of Medicine, Beth Israel Deaconess Medical Center, Boston, Massachusetts, USA; Department of Medicine, Harvard Medical School, Boston, Massachusetts, USA

**Keywords:** global epidemiology, HIV prevention, missed opportunities, narrative review, nonoccupational postexposure prophylaxis for HIV

## Abstract

Postexposure prophylaxis (PEP) is an important tool for preventing HIV infection but remains underutilized. In this narrative review, we aim to summarize the frequency of missed opportunities for prescribing PEP among studies from around the world, discuss the complexities of the challenges facing PEP provision, and describe possible solutions. We identified 20 studies published in the last 10 years among 43 832 individuals, of whom an estimated 41 477 were eligible for PEP. Of those eligible for PEP, PEP was prescribed among 27 705 (66.8%). There was a significant difference in PEP prescriptions in acute compared with non–acute care settings (63.5% vs 94.5%; *P* < .001). Emergent themes contributing to PEP underutilization included lack of provider and patient awareness, reduced PEP acceptability, HIV stigma and homophobia, lack of access (either to care or to medication), and stigmatizing policies. Each of those issues should be the focus of future PEP implementation efforts.

## Teaser

Post-exposure prophylaxis for HIV is underutilized globally. Drivers of that underutilization are multifactorial and interrelated, including patient- and provider-level barriers to PEP awareness and acceptability, as well as structural and systemic factors.

Postexposure prophylaxis (PEP) for HIV infection using antiretroviral medication is recommended as a key component of the strategy to combat the HIV epidemic by the US Centers for Disease Control and Prevention as well as the World Health Organization [1, 2]. PEP was first evaluated for use in humans as a means of preventing occupational exposures. While no randomized clinical trial data exist, observational studies have suggested that PEP is effective in preventing HIV acquisition from both occupational exposures, such as needle sticks, and nonoccupational exposures, such as condomless sex or after sharing injection drug use paraphernalia [3, 4]. However, PEP is notably underutilized [5].

An important component of PEP is that its effectiveness depends on the duration between exposure and initiation of therapy; consequently, PEP is only recommended within 72 hours of an exposure. That recommendation is based on early animal models [6] and extrapolated from postnatal prophylaxis using nevirapine [7]. That narrow window, however, necessitates patient and provider awareness of both PEP as an option and the need to promptly begin therapy. Additional barriers arise in contexts when PEP is indicated as a consequence of stigmatizing events that may limit health-seeking behaviors, such as after sexual assault and/or same sex intercourse. After successful initiation of PEP, completion of the 28-day course is another obstacle to maximal PEP effectiveness, either because of the daily pill burden, side effects (depending on the regimen), or lack of consistent access to medication in low-resource settings.

All of those factors have contributed to missed opportunities for PEP delivery; however, data are limited on the actual burden of missed PEP prescribing. Unlike metrics established for pre-exposure prophylaxis (PrEP), for example, the PrEP-to-need ratio [8], there are no similar heuristics to support tracking of secular PEP trends and measuring unmet needs. In this review, focusing primarily on nonoccupational exposures, we aim to describe the characteristics of individuals eligible for PEP, summarize the frequency of missed opportunities for prescribing PEP globally, discuss the complexities of the challenges facing PEP programs, and highlight possible solutions.

## THE UNMET NEED FOR PEP

To understand who is most likely to benefit from PEP, it is essential to identify those at greatest risk for HIV infection, which factors augment the risks for transmission, and to what extent some of those transmission events are preventable. In 2022, there were an estimated 1.3 million new diagnoses of HIV infection globally [9]. The median HIV prevalence among adult men who have sex with men (MSM) was 7.5%—it was 10.3% among transgender women—compared with 0.7% overall [9]. Additionally, in the United States, Black and Latinx MSM are disproportionately affected compared with White MSM; of the >32 000 new cases of HIV in the United States in 2021, 48% occurred among Black and Latinx MSM [10]. Subpopulations such as migrant MSM from low-resource areas living in high-income countries also experience disproportionately worse HIV outcomes, likely driven by decreased access to care and social stigma [11]. Other important risk factors for HIV acquisition include sex work, injection drug use, and incarceration [9, 12].

To what extent the 1.3 million new infections in 2022 were preventable using PEP remains unclear. Discrete high-risk events, such as sexual assault, constitute an example of when PEP might be most impactful. One study among a cohort of women exposed to rape documented an incidence rate of HIV acquisition of 6.6 per 100 person-years, equating to a 60% higher risk for acquiring HIV compared with those not exposed to rape [13]. Casual sex, venue-based sex, group sex, and transactional sex among serodiscordant partners are similarly high-risk events. Of the 39 million people with HIV globally in 2022, 3.5 million were unaware of their status [9], and thus potential transmitters of the infection. Even among those with HIV engaged in care, an estimated 29% of people globally remain virologically unsuppressed [9]. Prior work has estimated that >90% of HIV transmissions result from individuals either unaware of their HIV serostatus or not engaged in care (and thus virologically unsuppressed) [14]. Yet, even people with HIV who are regularly engaged in care can transmit the virus [15]. Therefore, clinicians and sex partners need to consider PEP as a potential prevention strategy. Moreover, the PEP encounter may provide an “educable moment” that can enable an informed provider to counsel patients about how to decrease future risks and that could lead to discussions regarding the use of pre-exposure prophylaxis [16].

## PEP UTILIZATION

In support of a narrative review of PEP utilization, we systematically searched PubMed for studies published in the last 10 years reporting on the proportion of patients presenting for and prescribed PEP among individuals with PEP indications (Supplementary Box 1). Of 155 articles, we identified 20, from 10 countries (Supplementary Figure 1), that provided data useful for our review (Supplementary References). Those 20 studies included 43 719 individuals, of whom an estimated 41 477 were eligible for PEP. Of those, 27 705 (66.8%) were prescribed PEP. PEP was prescribed among 63.5% compared with 94.5% of eligible patients in acute and non–acute care settings, respectively (*P* < .001, chi-square test). We defined PEP eligibility based on each individual study; it was relatively uniform across studies. However, 3 studies from acute care settings (n = 29 744 individuals accounting for 19 721 PEP prescriptions) [17–19], did not define PEP eligibility, and all individuals included in those studies were presumed PEP eligible in this analysis.

Table 1 shows the individual studies that included patients presenting to acute care settings (eg, emergency departments and specialized sexual violence clinics). Those studies included 38 355 individuals, with 37 040 (96.6%) classified as eligible for PEP, among whom 23 511 (63.5%) were prescribed PEP. Importantly, 2 of those studies included health care exposures (n = 491) that could not be distinguished from other exposures [17, 27]. Additionally, 3 of those studies included patients also presenting to nonacute settings (primary care, obstetrics clinics, HIV specialty clinics, and anonymous screening centers), but those data could not be disaggregated. The risk behaviors that constituted PEP eligibility in acute care settings were predominantly consensual sex (with a partner with HIV or unknown HIV status) and sexual assault, while 1 study included patients exposed via soiled needles, assault injuries, bites, scratches, and saliva [22] and 1 study included sex workers (n = 2) [27]. One study did not report the risk behaviors [24].

**Table 1.** Studies on Nonoccupational HIV Postexposure Prophylaxis Prescribing in Acute Care Settings

First Author's Last Name	Country	Year	Setting	Risk Factor	No. Included in the Study	No. Eligible for PEP	No. Offered PEP	No. Prescribed PEP	No. HIV Seroconversions	Reference
Silva-Nash	United States	2022	Emergency department	Sexual assault	147	133	118	96	0	20
Malinverni	Belgium	2016	Emergency department	Consensual sex (n = 1093), sexual assault (n = 209), other (n = 55)	1357	949	-	940^a^	1^b^	21
Rouveix	France	2014	Emergency department, anonymous screening centers	Consensual sex	633	633	-	486	0	22
				Consensual sex and other^c^	1139	1139	…	348	0	
				Other^c^	75	75	…	26	0	
Casalino	France	2019	Emergency department	Consensual sex (n = 1333), other (n = 200)	2011^d^	2011	-	974	-	17
Tapesana	Zimbabwe	2017	Emergency department	Sexual assault	474	129^e^	-	18	-	23
Casalino	France	2022	Emergency department	Nonoccupational (not otherwise specified)	200	-	-	133	-	24
Scholten	Germany	2018	University hospital	Consensual sex (n = 459), sexual assault (n = 25)	484	-	351	348	0	25
Nisida	Brazil	2019	Specialized sexual violence clinic	Sexual assault	197	167	-	160^f^	0	18
Schilling	United States	2017	Emergency department	Sexual assault	169	169	…	157^g^	-	26
Heck	Germany	2024	Emergency department	Consensual sex (n = 97), sex work (n = 2)	113^h^	66	-	65	-	27
Marzel	Switzerland	2017	Emergency department	Consensual sex	1051^i^	779	-	294	-	28
Gantner	France	2020	Emergency department, HIV specialty clinic	Consensual sex (n = 26 053), sexual assault (n = 3007)	29 060^i^	-	-	19 240	1^j^	19
Seidman	United States	2016	Emergency department, primary care clinics, obstetrics clinics	Consensual sex	27	7	4	2	0	29
Ebert	Germany	2018	Specialized sexual violence clinic	Sexual assault	1218	1168	-	224	0	30

Abbreviation: PEP, postexposure prophylaxis.

^a^An additional 7 individuals were prescribed PEP with a clear contraindication and were excluded from the analysis.

^b^One seroconversion reported condomless receptive anal intercourse with an HIV-positive source at the time of PEP initiation; subsequently completed PEP regimen without reporting successive risk behavior.

^c^Included discarded needle, soiled needle, assault injury, bite, scratch, and spit.

^d^Four hundred seventy-eight patients were health care workers who had an occupational exposure and were provided occupational PEP.

^e^Limited to only those who presented ≤72 hours after exposure.

^f^Three patients refused PEP; for 4 patients, there was no explanation provided for reasons not to prescribe PEP.

^g^Among the 157 prescribed PEP, 47 prescriptions were for the wrong duration and 5 included an incorrect or incomplete regimen.

^h^Thirteen patients were health care workers who had an occupational exposure and were provided occupational PEP.

^i^Number of visits for PEP (as opposed to patients).

^j^Occurred in a patient who stopped PEP after 2 days because of side effects and tested positive for HIV 3 weeks later.

Table 2 shows the individual studies that included patients presenting to non–acute care settings (primary care, HIV specialty clinics, and internet-based PEP provision services). Those studies included 5477 individuals, 4437 of whom were classified as PEP eligible, and 4194 (95.5%) were prescribed PEP. The HIV risk behaviors reported in those studies included consensual sex, intravenous drug use, and sexual assault. One study was conducted among individuals presenting for care after a sharp exposure in public settings [35].

**Table 2.** Studies on Nonoccupational HIV Postexposure Prophylaxis Prescribing in Primary and Specialty Care Clinic Settings

First Author's Last Name	Country	Year	Setting	Risk Factor	No. Included in the Study	No. Eligible for PEP	No. Offered PEP	No. Prescribed PEP	No. HIV Seroconversions	Reference
Braun	United States	2022	Primary care	IV drug use (n = 175), consensual sex (n = 47)	204	7	4	4	-	31
Shan	China	2023	Online	Consensual sex (n = 389), other (n = 150)	539	539	539	539	0	32
Thomas	Canada	2015	HIV specialty clinic	Consensual sex	3547	2883	-	2772	10^a^	33
Kouanfack	Cameroon	2019	HIV specialty clinic	Sexual assault (n = 224), consensual sex (n = 75)	299	209	-	184	-	34
Dias Costa	Brazil	2017	HIV specialty clinic	Sharp exposure in public	528	465	…	371	-	35
McDougal	United States	2014	HIV specialty clinic	Sexual exposure (n = 334; including n = 79 sexual assault), IV drug use (n = 13)	360	334^b^	-	324	4^c^	36

Abbreviations: IV, intravenous; PEP, postexposure prophylaxis.

^a^Nine patients reported high-risk behaviors following PEP initiation; 1 was considered a true PEP failure.

^b^Two cases considered possible PEP failures; 1 case tested positive 5 months after completing PEP, 1 tested positive 1 year after completing PEP.

^c^Two hundred seventy-seven PEP prescriptions deemed appropriate based on Centers for Disease Control and Prevention guidelines, but no data were available on 26 patients who were not prescribed PEP that presented ≤72 hours after exposure.

## OBSERVED THEMES OF BARRIERS TO PEP UPTAKE

No study directly evaluated barriers to PEP use. Several studies presumed a lack of provider awareness or comfort with PEP regimens in acute care settings, and sought to develop and deploy educational programs to augment correct identification of eligible patients and prescription accuracy [17, 26]. Patient-level factors were also discussed by several studies, including patient awareness and PEP acceptability among patients [28, 36]. While not directly assessed, cost was also discussed by several studies, noting the possible role of medication costs on decreased PEP acceptability among patients [36]. Finally, while not addressed directly, structural factors such as stigma and stigmatizing policies, as well as lack of access to medications, were noted to potentially impact PEP delivery.

### Provider-Level Barriers to PEP

It is possible that providers in acute care settings are less aware of PEP as an intervention than providers in nonacute settings, which included HIV specialty clinics; however, provider awareness likely remains a major barrier in all settings. One 2020 survey of providers in non–acute care settings in the United States found that 13% were unaware of PEP and 44% were aware of PEP but had never prescribed it [37]. Similar surveys from Tanzania and Ethiopia demonstrated that between 52% and 72% of health care providers had low knowledge of PEP [38, 39]. Some settings may see a higher proportion of patients eligible for PEP upon presentation (eg, settings that see survivors of sexual abuse), while other settings might see a relatively lower frequency of PEP-eligible patients; implementation strategies to increase awareness and identification of PEP-eligible patients must be tailored to each context.

Provider acceptability of nonoccupational PEP remains important but may be attenuated by HIV-related stigma [40]. Other drivers of low provider acceptability of PEP include false conceptions that PEP is associated with increased HIV risk-taking behaviors or an increased risk for HIV antiretroviral resistance [41]. While individuals eligible for PEP are more likely to engage in higher-risk sex practices than individuals not eligible, the use of PEP has not been shown to lead to any additional increase in risky behaviors [42]. Furthermore, modeling data using PrEP has provided evidence that the emergence of antiretroviral resistance due to peri-exposure prophylaxis is extremely rare [43].

In addition to PEP awareness and acceptability, provider comfort with PEP prescribing, including selecting the correct regimen and dosages, as well as arranging appropriate follow-up, may be more limited in acute care settings; Schilling et al. reported that among the 157 PEP prescriptions in the emergency department setting, 29.9% were for the incorrect duration [26].

### Patient-Level Barriers to PEP

Lack of patient awareness may limit presentation to care within the necessary time window after an exposure. A meta-analysis of PEP awareness among 12 579 MSM concluded that 40% were unaware of PEP [44]. Among 14 transgender women surveyed, less than half had heard of PrEP or PEP [45]. Among female sex workers in Kenya, only 49% were aware of PEP [46]. One study among Chinese persons who used drugs found that only 30% were aware of PEP [47].

Low patient acceptability of PEP is another important barrier. Among MSM in rural China, the acceptability of PEP was 57%, with higher acceptability associated with having social support and having been previously tested for HIV [48]. Not accepting PEP when offered among patients presenting after an acute traumatic episode (such as sexual assault, assault by a current or former sex partner, isolated anal and/or genital injury) has been associated with lack of encouragement by the health care provider for PEP initiation [49]. While numerous studies have assessed the acceptability of PrEP among people who inject drugs and sex workers, PEP acceptability is less well documented. Among 401 Chinese people who used drugs, 57% reported willingness to use PEP [47]. A study assessing PEP acceptability among Jamaican sex workers noted that PEP acceptability was 71% despite only 33% being aware of PEP as an intervention. In that study, acceptability was associated with recent sexual or client violence [50]. Similar results were seen among a cohort of sex workers in China [51].

### Structural Barriers to PEP

Systemic and structural factors that limit PEP use pose important barriers in many settings, particularly those with limited resources. Lack of access to centers where antiretroviral therapy is available is a major limitation in resource-rich and resource-limited settings [52, 53]. Further, the lack of a reliable supply of medication likely contributes to both unfamiliarity with medication regimens and the low usage of PEP by providers, as well as lower acceptability among patients, as has been seen with PrEP [53]. Medication stockouts also risk reducing the effectiveness of PEP as an intervention by making it more difficult to initiate within the requisite narrow time frame or to complete the 28-day course. Additionally, many settings where PEP may be most needed because of a high prevalence of HIV lack human rights protections for individuals who engage in same-sex relationships [54], thereby potentially limiting both health care­–seeking behavior and acceptability of PEP for fear of persecution.

In some settings, systemic inequities limit access to treatment services among racial and ethnic minorities and other marginalized populations. In the United States, despite representing ∼70% of new HIV infections [10], the Centers for Disease Control and Prevention estimates that only 11% of eligible Black individuals and 20% of eligible Latinx individuals were prescribed PrEP in 2021, compared with 78% of eligible White individuals [55]. Notably, data are not available for PEP uptake by different racial groups.

## FUTURE DIRECTIONS

The major barriers to appropriate PEP utilization include low provider and patient awareness and acceptability of PEP and HIV stigma, as well as homophobia, transphobia, binophobia, and systemic factors such as lack of access to care or medication availability, all of which are compounded by the narrow window after an exposure in which PEP is effective in preventing HIV acquisition. Thus, underpinning other prevention programs, increased access to PEP should be a priority. Forthcoming World Health Organization guidelines will hopefully emphasize this by providing strong recommendations that PEP be made available in community settings and provided by lay workers. To facilitate such recommendations, however, efforts are needed on several fronts.

First, increasing provider awareness of PEP will be essential. Given the narrow time window in which PEP is effective, even slight delays in offering therapy can mean the difference between PEP eligibility and ineligibility, as well as PEP efficacy. Implementing universal and standardized PEP education programs across a wide range of settings will be important given the heterogeneity of providers and settings in which PEP may be indicated. To that end, providing unambiguous guidance as to the optimal formulation to offer will also be important in overcoming provider uncertainties.

Similarly, increasing community awareness could have a multitude of positive effects. Increasing community-level PEP awareness may help to normalize the intervention, which might combat stigma. Further, increased patient awareness will likely translate into earlier presentations to care, increasing the proportion of individuals who present within the window of effectiveness. One study from Uganda found that PEP awareness was 52% upon enrollment in an intervention to integrate HIV prevention into an adolescent empowerment program; at follow-up, 92% of participants were aware of PEP [56].

Such interventions will need to be tailored toward the setting and population in which PEP is being prescribed. For example, survivors of sexual assault may experience post-traumatic stress disorder, and longitudinal evaluation of these individuals is warranted in order to understand the best practices for offering PEP in such circumstances. Similarly, the optimal implementation of PEP among survivors of sexual assault may differ if offered in an urgent care or emergency department compared with a student health center. Expansion of PEP implementation trials should be considered to specifically evaluate PEP delivery among survivors of sexual assault.

Reducing barriers to accessing PEP will also be essential for some populations. Providing a course of PEP to have on-hand, also known as PEP-in-Pocket, may reduce the time from exposure to initiation of medication and may be particularly useful for patients who experience low-frequency high-risk events or challenges in presenting to care [57]. Co-formulated regimens using integrase strand transfer inhibitors are well tolerated [58] and may simplify medication delivery. New regimens are also under investigation including long-acting injectable therapies, topical gels, and vaginal inserts [59], which might facilitate improved access and adherence to therapy. Formulations that are required to be prescribed in their original packaging might also limit practical mechanisms of medication adherence support (eg, pill boxes); drug manufacturers should consider provision of drug stability data where applicable to permit such adherence aids. Subsequent work assessing barriers due to costs will also be important within different geographic and sociocultural contexts.

However, even when initiated appropriately, PEP is not a panacea. Completion of the recommended 28-day PEP regimen has been estimated to be ∼57% among all comers [60]. Regimen selection is a universally important component of ensuring adherence, as some regimens are better tolerated than others. However, PEP completion varies by population. Among MSM, PEP completion rates have been estimated to be ∼67% [60], while among survivors of sexual assault, PEP completion rates may be as low as 40% [60]. Interventions to improve adherence will likely need to be tailored to specific populations.

To better understand whether PEP is being adequately and appropriately used, it will be important to establish validated metrics to track trends in PEP eligibility, PEP prescribing practices, and PEP adherence, similar to what has been done for PrEP [8]. Cohort studies should be established to determine the PEP need in various diverse populations and to calculate the rates of PEP uptake, as well as the frequency of use, adherence, and persistence, and the rates of transition to PrEP. The ultimate metrics that will determine the population-level effectiveness of PEP will be comparison of rates of its uptake and HIV secular trends before and after its introduction. Although it would be desirable to determine the direct effect of PEP among individuals in real-world settings, measuring this is not a simple undertaking. Surveys of electronic medical record data may provide some insights regarding the frequency of prescriptions of PEP, but generally will not provide sufficiently granular data to determine whether all individuals who might benefit from PEP were offered it, accepted it, and whether those who received a prescription used it whenever they had a relevant exposure. However, if established, such metrics will guide interventions to ensure that PEP is reaching the people who need it the most.

Additional areas of HIV prevention will also require ongoing support, such as serostatus awareness programs, which will permit individuals to triage their need for PEP more appropriately and to use other prevention methods (eg, condoms, PrEP, etc.). Additionally, individuals requiring PEP have been shown to be at high risk for HIV acquisition during a subsequent exposure, thus supporting the idea that linkage to PrEP remains vital [16]. Finally, actively combating structural barriers to PEP access, such as avoidance of antiretroviral deserts where no sources of PEP are available and challenging stigmatizing policies, is needed in order to maximize the efficacy of PEP's role in ending the HIV epidemic.

## LIMITATIONS

Our study has several limitations. First, most missed opportunities for PEP likely go unnoticed and unreported. The articles reviewed provide an imprecise estimate of the frequency with which PEP is not prescribed when indicated for several reasons. First, data were not available for many regions with the highest prevalence of HIV, where PEP is likely an important prevention strategy. Second, the articles reviewed included heterogeneous populations; for example, Braun et al. included a cohort of participants presenting to a substance use program (only 3% met PEP eligibility criteria) [31], while Marzel et al. included all persons presenting to the emergency department after consensual sex (74% eligible for PEP) [28]. The fact that the majority of the reviewed articles focused on PEP delivery may have introduced observer bias, resulting in better outcomes than might be seen in real-world settings. Therefore, the proportion of eligible patients for whom PEP was not prescribed may be an underestimation. Conversely, PEP is not always the optimal strategy to prevent all HIV exposures. PEP should not be the norm in every consensual relationship in areas with a high HIV prevalence. In that setting, efforts should focus on increasing serostatus awareness among sex partners, and if they are in a serodifferent relationship, the goals should be to ensure that the person with HIV has access to treatment and successful viral suppression and to ensure that the uninfected partner has access to and uses PrEP.

## CONCLUSIONS

PEP is effective but must be initiated soon after an exposure to be efficacious. The barriers to PEP initiation are numerous, including provider, patient, and structural factors, all of which differ among diverse subpopulations who are at risk for HIV. Overall, however, PEP is underutilized. This review highlights that a substantial percentage of patients eligible for PEP have not been receiving it, delineating barriers and describing potential solutions. Future work should focus on increasing provider and patient awareness and acceptability of PEP, overcoming stigma, ensuring adequate supply of antiretroviral medication, and challenging policies that have a detrimental effect on HIV prevention efforts. While novel formulations of PEP are in the pipeline, demonstrating noninferiority will be challenging. Thus, current efforts should focus on making PEP as widely accessible and adopted as possible.

## Supplementary Data

Supplementary materials are available at *Open Forum Infectious Diseases* online. Consisting of data provided by the authors to benefit the reader, the posted materials are not copyedited and are the sole responsibility of the authors, so questions or comments should be addressed to the corresponding author.

## Supplementary Material

ofae332_Supplementary_Data
