# Supplementary material for: Missed Opportunities: A Narrative Review on Why Nonoccupational Postexposure Prophylaxis for HIV Is Underutilized
Source: Open Forum Infect Dis. 2024 Jun 15;11(8):ofae332. doi: 10.1093/ofid/ofae332 (PMC11289484; doi:10.1093/ofid/ofae332)
Supplement: ofae332_Supplementary_Data [file ofae332_supplementary_data.docx]

**Appendix**

**Supplemental Box: Search Terms Used for Systematic Review of Studies on Missed PEP Opportunities, October 30^th^, 2023**

| ("Practice Patterns, Physicians'"[Mesh] OR "Prescriptions"[mesh:noexp] OR "Drug Prescriptions"[mesh] OR prescrib*[tiab] OR prescription*[tiab] OR "physician decision"[tiab:~2] OR "physician's decision"[tiab:~2] OR "physician decisions"[tiab:~2] OR "physician's decisions"[tiab:~2] OR "physicians decisions"[tiab:~2] OR "physicians decisions"[tiab:~2]) AND ("Post-Exposure Prophylaxis"[Mesh] OR post-exposure prophylax*[tiab] OR post-exposure prevent*[tiab] OR post-exposure prescrib*[tiab] OR postexposure prophylax*[tiab] OR postexposure prevent*[tiab] OR postexposure prescrib*[tiab] OR PEP[tiab]) AND ("Sexually Transmitted Diseases"[mesh] OR "HIV"[mesh] OR "HIV infections"[mesh] OR sexually transmi*[tiab] OR hiv[tiab] OR human immmunodeficiency[tiab] OR human immmune deficiency[tiab]) AND (y_10[Filter]) |
| --- |

**Supplemental Figure: Map of Countries from which Studies on Missed PEP Opportunities were Included**

**
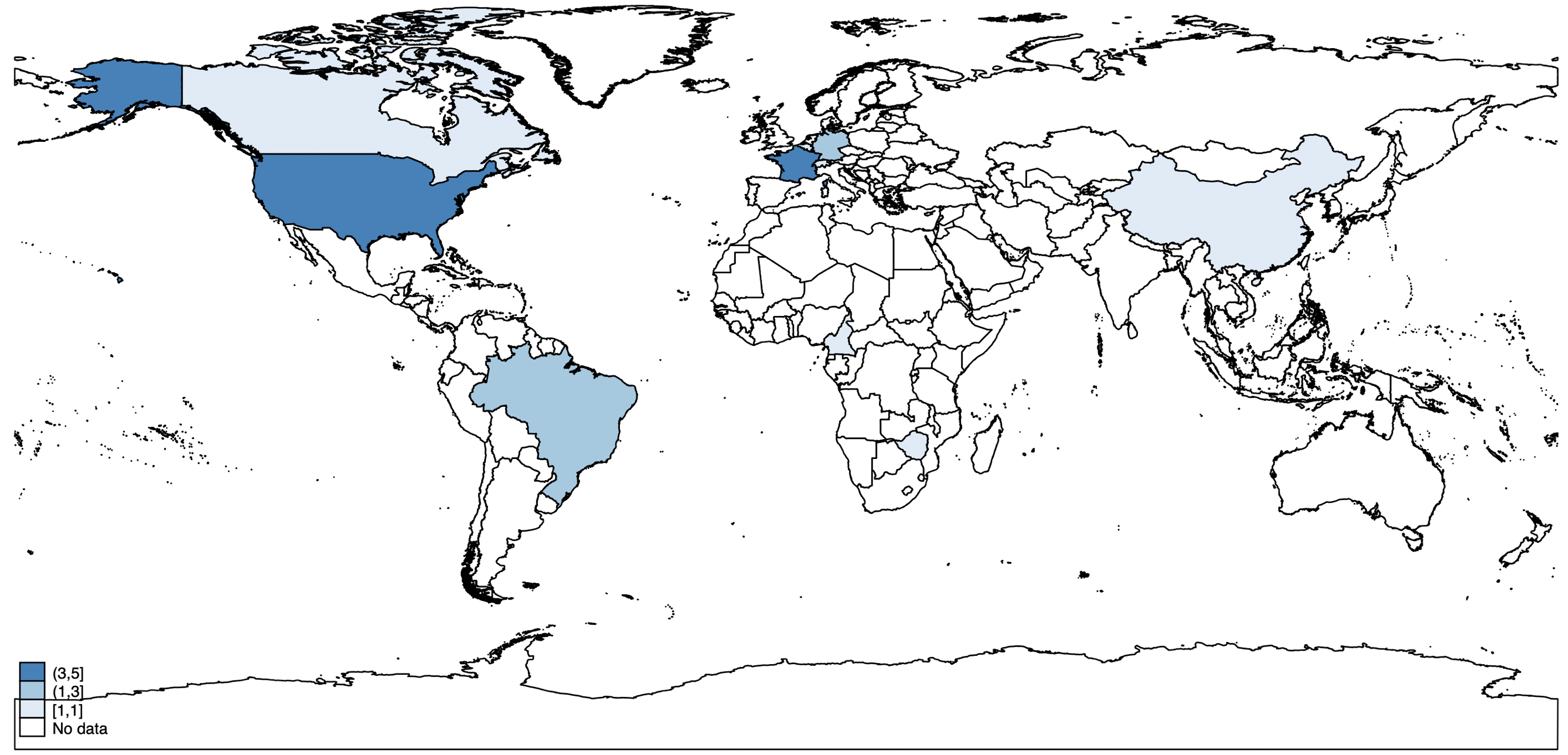
**
